# W135 Meningococcal Disease in Africa^1^

**DOI:** 10.3201/eid0911.020727

**Published:** 2003-11

**Authors:** Andrew J. Pollard, Maria Santamaria, Martin C.J. Maiden

**Affiliations:** * University of Oxford, United Kingdom; † World Health Organization, Geneva, Switzerland

Epidemic meningococcal disease has occurred in Africa for approximately 100 years and has been recognized as a particular problem in sub-Saharan Africa, “the meningitis belt,” since 1963. Despite intervention with plain polysaccharide vaccines, thousands of cases and deaths continue to occur. The circumstances that have driven these epidemics of disease remain poorly understood, but a number of factors are likely to be important, including crowded living conditions, population movements, seasonal factors, and the characteristics of the meningococci circulating at a given time. During the latter half of the 20th century, serogroup A meningococci have been responsible for most epidemic disease in Africa; however, as with other regions of the world, cases caused by serogroup B, C, Y, W135, and X meningococci have been occasionally responsible for epidemics. Some epidemic disease outbreaks have been associated with the annual Hajj pilgrimage (e.g., the spread of serogroup A meningococci during the late 1980s and the spread of W135 meningococci from 2000 onwards). Mass vaccination with serogroup A/C plain polysaccharide vaccines has been used to control outbreaks, once they have been identified, in a number of African countries. However, the efficacy of this reactive approach has been questioned, and the recent occurrence of W135 epidemics, combined with a global shortage of the polysaccharide vaccines, creates renewed urgency for a rational and universal preventive program.

This workshop explored the scientific issues behind the design and implementation of a vaccine strategy for the meningitis belt of Africa focusing on the epidemiology of meningococcal isolates. Epidemiologic studies have provided an increasingly detailed knowledge of meningococcal disease in Africa. This knowledge has led to the identification of three distinct clonal complexes responsible for serogroup A disease in Africa (ST-1 complex, ST-4 complex, and ST-5 complex) with successive large-scale epidemics caused by ST-1 and ST-5 complex. Recent epidemiologic findings have shown that serogroup A meningococci belonging to the ST-5 complex (ST-5 and ST-7) were still responsible for most cases and outbreaks of disease in 2000, 2001, and 2002, with the W135 epidemics caused by bacteria belonging to the ST-11 complex. This complex has previously been associated with serogroup C disease. However, while knowledge of the clonal complexes has provided important information on meningococcal disease in Africa, more detailed isolate characterization has shown that important diversity is overlooked by relying solely on sequence type. Despite the availability of a number of meningococcal typing strategies (including pulsed-field gel electrophoresis, multilocus enzyme electrophoresis, and 16s rRNA typing), to date, no portable method is broadly accepted for identifying subvariants below the level of clonal complex. Funding for fundamental research to improve methods of analysis of diversity and dynamics of these populations is an urgent requirement.

Since 2000, serogroup W135 meningococci (ST-11) have been isolated from sporadic cases in Algeria, Cameroon, Chad, Senegal, Niger, and Central African Republic and at the end of a serogroup A outbreak in 2001 and during a large outbreak during 2002 in Burkina Faso. Carriage studies demonstrated a high rate of carriage of W135 in some affected communities in Burkina Faso. Serogroup X has also been widespread in Africa (1970–2000) from studies in Mali, Niger, Burkina Faso, and Ghana. Serogroup X has been primarily found in healthy carriers but also in occasional epidemics.

These studies highlight the importance of supporting enhanced laboratory surveillance throughout the region to monitor the spread of nonserogroup A meningococci. Polymerase chain reaction may increase case ascertainment, but basic microbiologic testing on a large scale is required.

Several studies have been performed on carriage isolates from pilgrims returning from the Hajj. Since 2000 and the introduction of ST-11 complex, W135 meningococci among carried isolates in North Africa (Sudan, Morocco) was documented. By contrast, despite a small increase in cases associated with the Hajj, rates of disease caused by ST-11 W135 meningococci in Europe remained low since 2000, with some evidence that most activity was limited to the Muslim communities.

One study found that the minority (8%) of W135 (case and carrier) isolates are *O-*acetylated (*O*ac+) in the United Kingdom and that the currently available tetravalent polysaccharide vaccine evokes bactericidal activity against both *O*ac+ and *O*ac- W135 and Y isolates. The relevance of *O-*acetylation to vaccine development remains uncertain.

To plan intervention strategies in Africa, changes in the major vaccine antigen (the capsular polysaccharide) present among epidemic disease isolates should be closely monitored. Fundamental research to understand diversity and dynamics of these important bacterial populations is required. The recent epidemic of W135 and substantial numbers of cases caused by other non-A serogroups (X and C particularly) provide uncertainty about the future epidemiology of capsule expression during epidemics. Epidemic meningococcal disease in Africa might no longer be thought of as a peculiarity of serogroup A meningococci. The central idea from the workshop was that a comprehensive vaccine (i.e., a multivalent-conjugate) was the optimal approach to controlling epidemic disease in the meningitis belt of Africa. Even this approach may fail, given the remarkable adaptability of this variable organism. Further outbreaks of W135, as well as serogroup A, might occur in the region, and contingency planning for control of W135 outbreaks is required.

A sustainable vaccine program for Africa is needed to prevent future epidemics. Conjugate vaccines provide the possibility of generating protective immunity from infancy and ending epidemic disease. Such vaccines have now been developed by pharmaceutical companies in industrialized nations. However, the challenge is the delivery of effective and affordable vaccines in sub-Saharan Africa, which has not so far been possible in collaboration with major pharmaceutical manufacturers. Practical and economic difficulties exist in delivering an affordable tetravalent ACYW conjugate vaccine for Africa, which, as outlined above, is an important objective. The Meningitis Vaccine Project will support the development of an affordable monovalent serogroup A conjugate polysaccharide vaccine in partnership with a developing country manufacturer. In the long term, this approach allows the possibility of sustainable prevention of epidemics in the region and is of great importance. This workshop concluded that a monovalent serogroup A strategy could leave the population exposed to the risk for further non-A epidemics and that strategies that include other serogroups, particularly W135, need to be put in place as soon as is possible.

Development of an affordable vaccine for Africa cannot be achieved quickly. Discussion of the urgent issue of vaccines for control of epidemics of meningococcal disease in the next few years was not possible during the workshop. The current polysaccharide vaccine shortages raise the possibility that epidemic meningococcal disease continue with no intervention available. ACYW-conjugate vaccines are in development by several major vaccine manufacturers; however, without a market to drive production of millions of doses for sub-Saharan Africa, many more people might die before an affordable vaccine can be delivered by the Meningitis Vaccine Project.

